# 

**Published:** 1828-05

**Authors:** 


### TO SUBSCRIBERS, CORRESPONDENTS AND EDITORS.

1. It will be recollected that our numbers are to consist of 56 and 48 pages alternately. On this plan the present number would comprehend 48 only. As a considerable part of the Discourse on Intemperance may not, however, be regarded as sufficiently professional, we have extended the number eight pages beyond its proper limits, which, we hope. will satisfy all who are interested.

2. The third number, which will be put to press immediately, will contain Original Essays and Cases, by Dr. Coons, of Alabama, Dr. Brower, of Ohio, and the Editor.

3. The following Journals have been received, in exchange :—

The American Medical Recorder, for April, 1828.

The American Journal of the Medical Sciences, for May.

The North American Medical and Surgical Journal, for April.

The New-York Medical and Physical Journal, for January, February and March.

The American Journal of Science and the Arts, for the same period.

The Transylvania Journal of Medicine and the Associate Sciences, Nos. 1 and 2.

The Boston Medical and Surgical Journal, (weekly) from No. 2 to 14—(1st No. miscarried.)

